# A two‐center randomized controlled trial of a repairing mask as an adjunctive treatment for mild to moderate rosacea

**DOI:** 10.1111/jocd.16413

**Published:** 2024-06-03

**Authors:** Liwei Wang, Yiyi Zhang, Lihong Chen, Dengfeng Yuan, Xiamei Feng, Jie Zheng, Xia Li, Chao Yuan

**Affiliations:** ^1^ Skin and Cosmetics Research Laboratory Skin Disease Hospital of Tongji University Shanghai China; ^2^ Department of Dermatology, Ruijin Hospital Shanghai Jiao Tong University School of Medicine Shanghai China; ^3^ Shanghai Jahwa United Co., LTD Shanghai China

**Keywords:** adjuvant therapy, minocycline, repairing facial mask, rosacea, skin barrier

## Abstract

**Objective:**

To investigate the efficacy and safety of a repairing mask as an adjunctive treatment for skin barrier maintenance of mild to moderate rosacea.

**Methods:**

Patients with rosacea were recruited in this dual center randomized controlled trial from November 2019 to December 2021. A total of 64 patients were included and randomized into two groups at a ratio of 3:1 into a mask group (*n* = 47) and a control group (*n* = 17). Patients in the mask group received treatment with Dr. Yu *Centella asiatica* repairing facial mask three times weekly for a duration of 6 weeks. All participants were instructed to continue their regimen of 50 mg oral minocycline twice daily and to apply Dr. Yu Intensive Hydrating Soft Cream twice daily. The primary endpoint of this study was the Investigator Global Assessment (IGA) score.

**Results:**

A total of 54 patients completed this trial, with 41 in the mask group and 13 in the control group. After using this facial mask for 3 and 6 weeks, the IGA, facial skin dryness, facial flushing, and severity of skin lesion in the mask group showed significantly improvement (*p* < 0.05). Moreover, the change in the delta degree of skin flushing was significantly higher than that in the control group (*p* = 0.037). Throughout the study, no adverse events were reported in either group of participants.

**Conclusion:**

The Dr. Yu *Centella asiatica* repairing facial mask, as an adjunctive treatment of rosacea, appears to effectively repair and protect the skin barrier, alleviate cutaneous symptoms of rosacea, and is both efficacious and safe for patient use.

Rosacea represents a prevalent chronic inflammatory dermatological condition characterized by compromised facial skin barrier integrity. Epidemiological evidence indicates a global mean prevalence of 5.46%.^1^ The clinical spectrum of rosacea is marked by recurrent flushing, transient or persistent erythema, and may include associated manifestations such as edema, papules, pustules, and telangiectasia.^2^ The therapeutic approach to rosacea encompasses general dermatological care complemented by both topical and systemic pharmacotherapy.^2^ Numerous national and international clinical guidelines consistently underscore the significance of evidence‐based skincare practices in the restoration of the skin barrier, alleviation of clinical manifestations, and extension of remission intervals.^3^, ^4^ Prior research has substantiated that numerous skin repair moisturizing formulations markedly ameliorate skin barrier function and augment treatment outcomes in patients with dermatological conditions.^5^ However, empirical evidence remains scarce regarding the utility of facial mask products in routine skincare regimens for individuals with rosacea, particularly as a modality for skin barrier restoration and defense to support rosacea management. This present investigation is pioneering in its examination of the therapeutic potential and tolerability of a reparative facial mask when employed as a complementary treatment in the rosacea patient cohort.

## 1 OBJECTS AND METHODS

### 1.1 Research participants

This investigation constitutes a bicentric randomized controlled trial (RCT) recruiting rosacea patientsat at two hospitals in Shanghai, conducted from January 2021 to December 2021.

Inclusion Criteria:

1. Female participants, healthy, aged between 25 and 60 years;
2. A clinical diagnosis of mild to moderate rosacea, with a Rosacea Investigator Global Assessment (IGA) score ≥3 points;
3. Provision of informed consent prior to study enrollment;
4. Absence of significant cardiac, hepatic, renal, gastrointestinal, neurological, psychiatric, or metabolic disorders;
5. No use of relevant pharmacological treatments within 1 month prior to the study onset and no topical antibiotics within the preceding week.

Exclusion criteria:

1. Current pregnancy, plans to become pregnant, or breastfeeding;
2. Documented hypersensitivity to any active ingredients or excipients contained in the study medication, or a history of adverse reactions to cosmetic products;
3. Enrollment in other clinical studies within 3 months prior to the initiation of the current trial;
4. Regular use of traditional Chinese medicine, hypnotics, sedatives, anxiolytics, or substances with potential for addiction;
5. Individuals with alcohol dependency or those unable to comply with medical recommendations;
6. Presence of concomitant dermatological conditions that may confound the clinical evaluation of acne or pose a risk to the participant, or a significant history of serious dermatological disorders;
7. Any additional factors deemed by the investigator to preclude participation in the study.

This study has been approved by the Ethics Committee of each site [Approval No. (2019) Clinical Ethics Review No. (59)]. Informed consent was obtained from all study subjects prior to enrollment.

### 1.2 Study method

#### 1.2.1 Test method

Participants were instructed to cleanse their face with warm water subsequent to providing informed consent. Clinical evaluations were conducted following a 30‐min acclimatization period in a controlled environment with stable temperature and humidity. This included a self‐assessment questionnaire and an investigator‐led evaluation of clinical symptoms. The aforementioned procedures were repeated at 3‐week and 6‐week intervals post‐product application.

#### 1.2.2 Randomization and blinding

According to the random number table, subjects were randomly assigned to either the test group or the control group at a 3:1 ratio. All assessments were conducted by clinicians who did not participate in the visits and were instructed not to communicate with investigators or subjects involved in the visits, and all clinicians were required to be trained in standardized clinical scoring criteria.

#### 1.2.3 Methods of administration

Participants in the test group applied Dr. Yu Intensive Hydrating Soft Cream twice daily and Dr. Yu *Centella asiatica* repairing facial mask thrice weekly for a duration of 6 weeks. Control group participants applied Dr. Yu Intensive Hydrating Soft Cream exclusively twice daily. All participants were administered 50 mg of oral minocycline twice daily for 6 weeks. In the event of a serious adverse reaction, the medication regimen may be discontinued. Participants were prohibited from using additional rosacea treatments or any agents that could potentially affect the evaluation of the study outcomes.

#### 1.2.4 Study endpoints

The primary endpoint was the Investigator's Global Assessment (IGA) score, while secondary endpoint encompassed skin dryness, skin flushing intensity, lesion severity, and participant‐reported assessments.

#### 1.2.5 Assessment of clinical symptoms

Patients were evaluated for the severity of rosacea utilizing the IGA score. Skin dryness was evaluated as follows: a score of 0, indicative of no evidence of dryness, was characterized by smooth skin; a score of 3, indicative of mild dryness, was characterized by patchy desquamation; a score of 6, indicative of moderate dryness, was characterized by uniformly distributed desquamation with fine scaling, yet not extensive; a score of 9, indicative of severe dryness, was characterized by pronounced scaling with widely spaced scales and the presence of chapped. The assessment of cutaneous flushing was similarly graded: score 0 = no evidence of flushing; score 3 = mild redness; score 6 = moderate redness; score 9 = very severe redness. Severity scoring criteria for skin lesions are shown in Table 1.

**TABLE 1:** Skin lesion severity scale.

Score	Erythema	Capillary dilatation	Number of papules and pustules	Lesion size
0	None	None	None	None
1	Mild	Mild	0–10	<10%
2	Moderate	Moderate	10–20	10%–30%
3	Severe or purplish	Severe	>20	>30%

#### 1.2.6 Subject self‐assessment

Self‐assessment refers to the assessment of skin symptoms, including tingling, burning, heat sensitivity, tightness, itching, pain, overall discomfort, and hot flashes; the scoring criteria are 0–10 points, 0 = none, and 10 = intolerable.

### 1.3 Statistical analysis

Statistical analysis was conducted using SPSS Statistics 22.0 software. Measurement data were calculated for mean and standard deviation, adhering to the normal distribution. For comparing product effects before and after use, a paired *t*‐test was employed. An independent samples *t*‐test was utilized for comparisons between the test group and the control group, while a rank sum test was applied to data not conforming to normal distribution. The rank sum test for related samples was used to compare ordinal data before and after use, and the rank sum test for independent samples was used for comparisons between the test group and the control group. Statistical significance in this study was determined using two‐tailed tests with *p* < 0.05 as the threshold for judging significant differences.

## 2 RESULTS

### 2.1 General information

In this study, a cohort of 64 subjects was initially recruited, of which 54 patients with confirmed rosacea met the eligibility criteria and successfully completed the trial (refer to Figure 1). The age ranged from 25 to 55 years with a mean of 38.16 ± 8.46 years. Attrition was observed in 10 subjects who were lost to follow‐up, attributable to their inability to attend scheduled visits due to restrictions imposed by the COVID‐19 pandemic.

**FIGURE 1:**
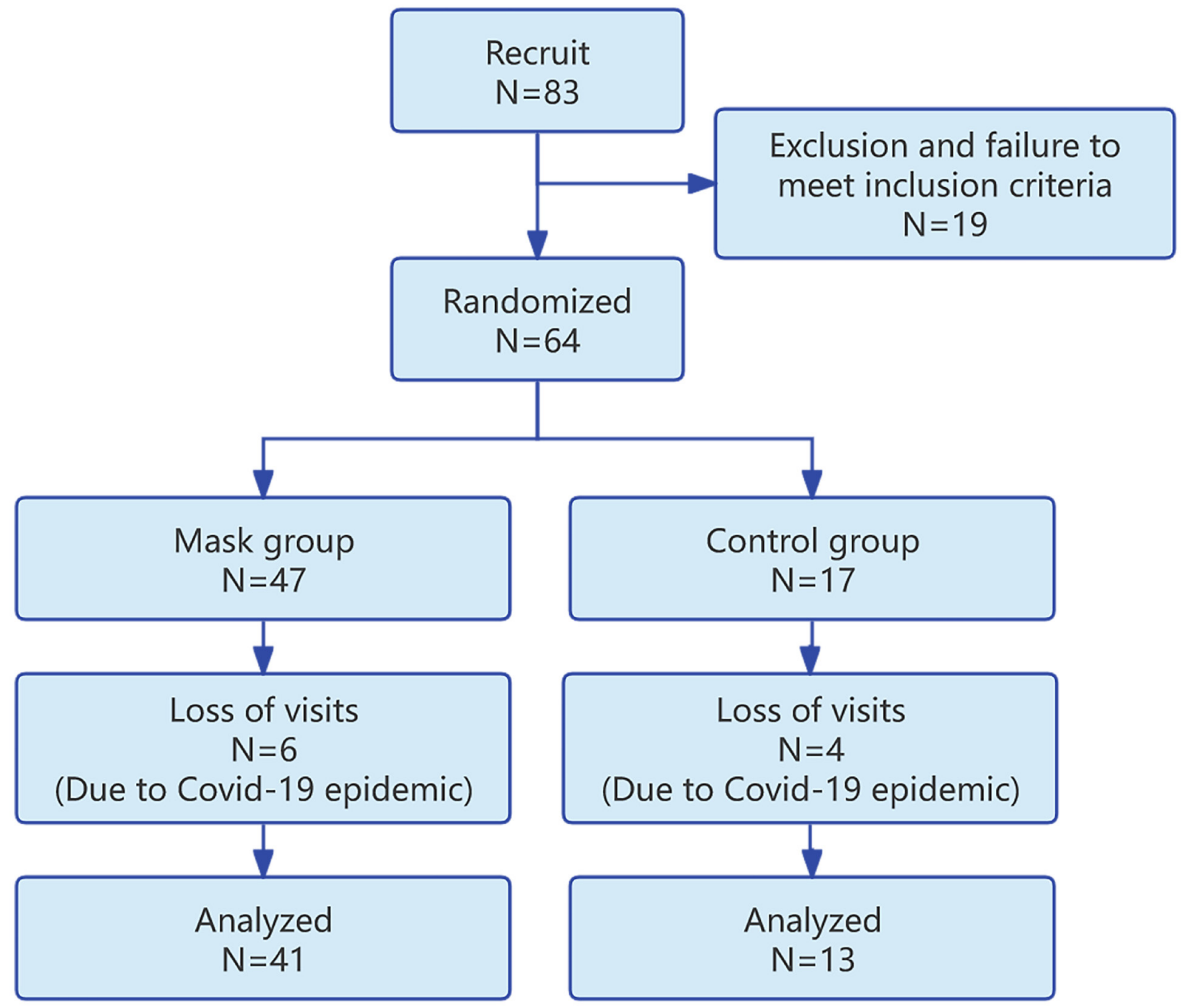
Study design.

### 2.2 Clinical assessment

The clinical evaluation outcomes are presented in Figure 2. Relative to baseline measurements, there were significant reductions observed in the Investigator's Global Assessment (IGA) score for facial rosacea, facial skin dryness score, skin flushing score, and severity score of facial skin lesions in both the test and control groups at 3 and 6 weeks (*p* ≤ 0.001). By the conclusion of the 6‐week period, the improvement in skin flushing in the test group was significantly greater than that in the control group, with a statistically significant difference (*p* = 0.037). The comparative image of the patient's facial red area can be seen in Figure 3.

**FIGURE 2:**
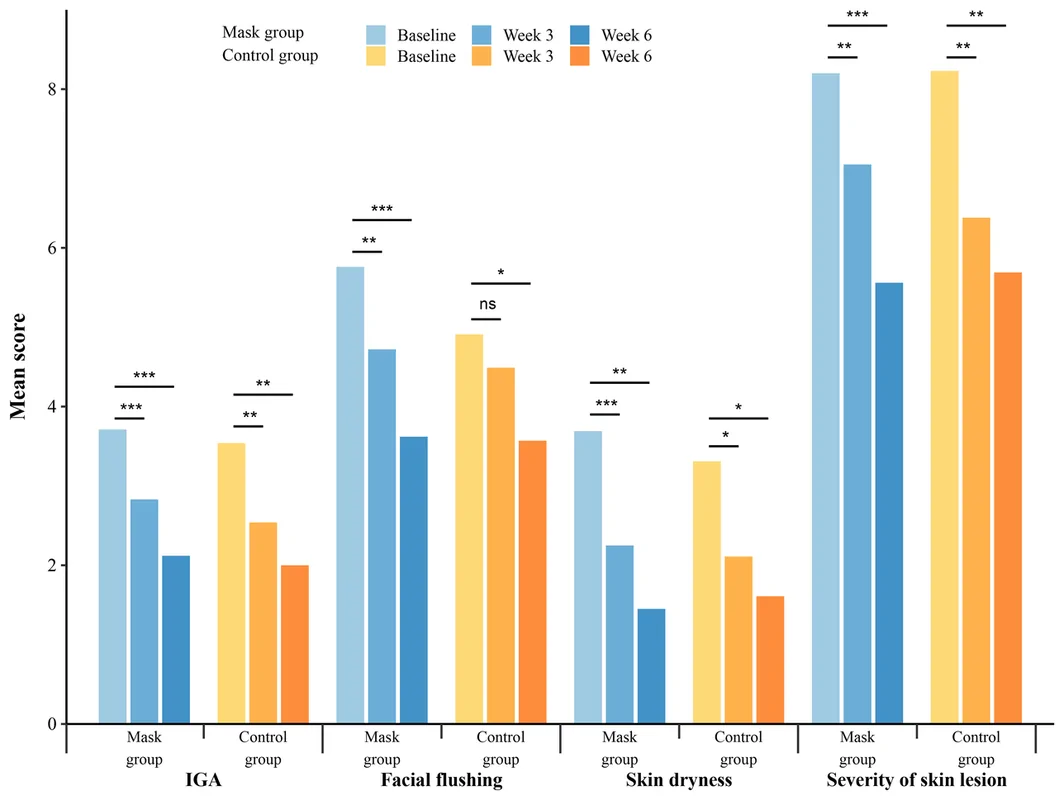
Dry skin, flushing, IGA and lesion severity scores. Statistically different from basal values, *p < 0.05, **p < 0.01, ***p < 0.001.

**FIGURE 3:**
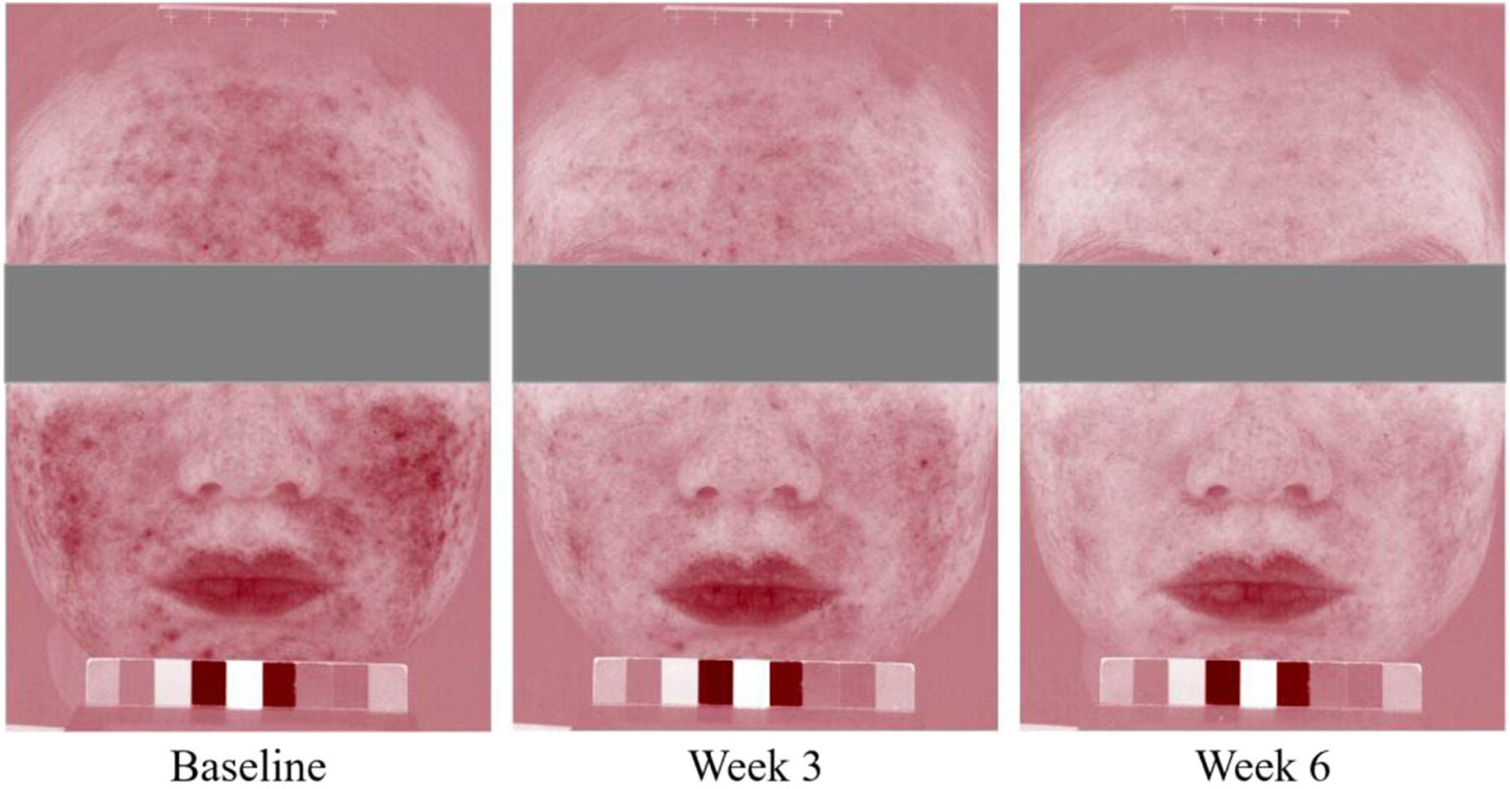
The comparative image of the patient's facial red area.

### 2.3 Patient self‐assessment

In comparison to baseline measurements, the test group exhibited a significant reduction in scores for burning sensation, heat sensitivity, tightness, itching, pain, and overall discomfort at both the 3‐week and 6‐week intervals, with the observed differences reaching statistical significance (*p* < 0.05). In the control group, significant decreases in scores for burning sensation, heat sensitivity, pain, and overall discomfort were observed at the 3‐week mark. By the 6‐week mark, the test group showed significant reductions in scores for burning sensation, heat sensitivity, itching, pain, and overall discomfort, with these differences also being statistically significant (*p* < 0.05). However, the severity scores between the test group and control group at various time points did not demonstrate a significant difference (*p* > 0.05), as detailed in Table 2.

**TABLE 2:** Severity scores of skin symptoms.

	Baseline		3 weeks		6 weeks	
	Control group	Mask group	Control group	Mask group	Control group	Mask group
Paresthesia	0 (0,6)	1 (0,9)	1 (0,4)	1 (0,7)	0 (0,3)	1 (0,10)
Burning sensation	5 (0,8)	5 (0,9)	2 (0,5)*	2 (0,10)***	2 (0,4)*	1 (0,10)***
Heat sensitivity	7 (1,9)	6 (0,9)	5 (2,9)	3 (0,10)***	2 (0,8)**	2 (0,10)***
Tightness	3 (0,10)	5 (0,9)	3 (0,4)	2 (0,7)***	2 (0,6)	2 (0,9)***
Itching	3 (1,9)	2 (0,10)	3 (0,5)	2 (0,10)*	2 (0,6)*	1 (0,10)**
Pain	1 (0,6)	1 (0,9)	0 (0,3)*	1 (0,7)*	0 (0,3)*	1 (0,9)*
Overall discomfort	5 (1,10)	5 (0,10)	3 (0,5)**	2 (0,9)***	2 (0,6)**	2 (0,9)***

*Note*: Statistically different from base values,*p < 0.05, **p < 0.01, ***p < 0.001; statistically different from controls, #*p* < 0.05.

### 2.4 Safety

No adverse events were reported in either group during the course of this study.

## 3 DISCUSSION

The current evidence indicates that impaired skin barrier function plays a significant role in the development and progression of rosacea. Individuals with rosacea have compromised skin barrier integrity and increased skin sensitivity, leading to a higher frequency of abnormalities such as parakeratosis, honeycomb structures, cavernous edema, and papillary dermal hyperplasia. These abnormalities are more prevalent in individuals with rosacea than in healthy controls, as observed using skin confocal microscopy.^6^ Therefore, restoring the skin barrier is a critical therapeutic approach for managing and relieving rosacea symptoms.

Clinical evidence has shown that effective skincare products aimed at repairing the skin barrier can not only alleviate symptoms of sensitive skin, such as dryness, tingling, and burning but can also reduce clinical signs like episodic flushing.^3^, ^4^

The Dr. Yu *Centella asiatica* repairing facial mask incorporates *Centella asiatica* extracts. Research has demonstrated that *Centella asiatica* possesses efficacious properties that facilitate tissue regeneration and repair damage,^7^ which is achieved through the stimulation of fibroblast proliferation and the synthesis of collagen.^8^ Furthermore, the extract of *Centella asiatica* is abundant in a multitude of natural bioactive compounds, notably saponins, flavonoids, and phenolic acids, which are known for their antioxidant and anti‐inflammatory properties. These constituents contribute to the enhancement of stratum corneum hydration, reduction of erythema, and amelioration of epidermal barrier function, thereby serving roles in hydration, moisturization, soothing inflammation, and barrier restoration.^9^ Asiaticoside, a principal constituent of *Centella asiatica* extract, is known to have anti‐inflammatory, antioxidant, and wound‐healing effects, making *Centella asiatica* a promising ingredient in skincare products. Clinical studies have provided evidence supporting the efficacy of asiaticoside in dermatological treatments. These studies have demonstrated its ability to improve wound healing, reduce scar formation, and alleviate inflammatory skin conditions. Furthermore, *Centella asiatica* has been shown to enhance the effectiveness of other skincare treatments when used in combination.^10^, ^11^ Additionally, the formulation of this facial mask incorporates trehalose, which functions as a prebiotic component, and has been shown to have moisturizing, antioxidant, and protective properties, making it a promising ingredient in skincare products. Research has demonstrated that prebiotics are capable of modulating the balance of cutaneous microflora^12^ and restoring impaired skin barrier function, consequently ameliorating symptoms associated with skin sensitivity.^13^ The pathophysiological manifestations of rosacea have been posited to be intricately linked with cutaneous dysbiosis.^14^ Consequently, the inclusion of *Centella asiatica* and prebiotic constituents in this mask may confer benefits in the alleviation and restoration of sensitive skin, correction of cutaneous microecological imbalances, reinforcement of the epidermal barrier, and promotion of skin self‐repair mechanisms.

To investigate the adjunctive therapeutic effects of Dr. Yu *Centella asiatica* repairing facial mask for the treatment of rosacea, subjects in the intervention group were administered Dr. Yu *Centella asiatica* repairing facial mask alongside minocycline and Dr.Yu Intensive Hydrating Soft Cream.

The findings indicated a significant reduction in the Investigator's Global Assessment (IGA) score for rosacea in the intervention group at both 3 and 6 weeks post‐treatment initiation with the facial mask, and comparative analyses demonstrated a numerically greater decrease in IGA scores within the intervention group than the control group at the 3‐week assessment point. Additionally, the study evaluated participants for cutaneous sensitivity, revealing a more pronounced decrease in sensitivity among subjects in the intervention group relative to those in the control group.

Additionally, the assessment of particular clinical manifestations, comprising skin dryness, facial erythema, and lesion severity, demonstrated a significant reduction in the test cohort's scores pertaining to dryness of facial skin xerosis, intensity of facial erythema, and lesion severity.

Collectively, these findings suggest that adjunctive therapy with the reparative mask, alongside antibiotic treatment, can efficaciously enhance the cutaneous barrier integrity in patients and ameliorate symptoms of skin hypersensitivity.

Compared to reparative moisturizing creams, facial mask formulations demonstrate superior conformity to skin contours. The humid microenvironment maintained beneath the facial mask during application enhances the penetration and absorption of active ingredients. Due to limitations in objective conditions, non‐invasive skin testing and the collection of biological samples were not conducted in this study. However, future research will address these aspects. This study's findings corroborate the efficacy of intermittent facial mask usage with hydrating properties. Furthermore, the absence of adverse reactions throughout the treatment period contributes to a favorable safety profile. While oral antibiotics are efficacious in ameliorating rosacea symptoms, adjunctive use of appropriate skincare products may further enhance cutaneous recovery, concurrently improving patients' quality of life and fostering greater confidence and adherence to ongoing treatment regimens.

In conclusion, this study provides initial evidence that Dr. Yu's *Centella asiatica* repairing facial mask offers a therapeutic benefit and is well‐tolerated when used as an adjunct therapy in patients with mild to moderate rosacea.

## AUTHOR CONTRIBUTION

The authors confirm that each author has participated sufficiently in the work to take public responsibility for the content, including participation in the concept, design, analysis, writing, or revision of the manuscript. The research was conceptualized and designed by Dr. Xia Li and Dr. Chao Yuan, who also facilitated data acquisition and provided the necessary funding and resources. Data collection, curation, and analysis were carried out by Dr. Liwei wang, Dr. Yiyi Zhang, and Dr. Lihong Chen, who were also responsible for the initial drafting of the manuscript, as well as its subsequent revisions. Dr. Jie Zheng contributed to the design of the study and the collection of data, in addition to participating in the critical review and editing of the manuscript. Dr. Dengfeng Yuan and Dr. Xiamei Feng are responsible for coordinating the project and preparing the test products. The final manuscript was read and approved by all contributing authors.

## ETHICS STATEMENT

This study has been approved by the Ethics Committee of each site [Ruijin Hospital, Shanghai Jiao Tong University School of Medicine, Approval No. (2019) Clinical Ethics Review No. (59)]. Informed consent was obtained from all study subjects prior to enrollment.

## CONSENT PROCEDURES

Informed consent was obtained from all individual participants included in the study. Additional consent was obtained for any individual persons' data in any form (including any individual details or images).

## ADDITIONAL INFORMATION ON RESEARCH PARTICIPANTS

This investigation constitutes a bicentric randomized controlled trial (RCT) recruiting rosacea patients at Shanghai Ruijin Hospital and Shanghai Dermatology Hospital, conducted from January 2021 to December 2021.

## PHOTO CONSENT

Written informed consent for publication of their photographs was obtained from the patients/subjects. A copy of the consent form is available for review by the Editor of this journal.

## Data Availability

The data that support the findings of this study are available on request from the corresponding author. The data are not publicly available due to privacy or ethical restrictions.
